# *In vivo* measure of neonate brain optical properties and hemodynamic parameters by time-domain near-infrared spectroscopy

**DOI:** 10.1117/1.NPh.4.4.041414

**Published:** 2017-08-18

**Authors:** Lorenzo Spinelli, Lucia Zucchelli, Davide Contini, Matteo Caffini, Jacques Mehler, Ana Fló, Alissa L. Ferry, Luca Filippin, Francesco Macagno, Luigi Cattarossi, Alessandro Torricelli

**Affiliations:** a CNR, Istituto di Fotonica e Nanotecnologie, Milano, Italy; b Politecnico di Milano, Dipartimento di Fisica, Milano, Italy; c SISSA, Language, Cognition, and Development Laboratory, Trieste, Italy; d CNRS-EHESS-ENS, Laboratoire de Sciences Cognitives et Psycholinguistique, Paris, France; e Presidio Ospedaliero Universitario “Santa Maria della Misericordia,” Neonatology Unit, Udine, Italy

**Keywords:** time-domain near-infrared spectroscopy, newborn, brain optical properties

## Abstract

By exploiting a multichannel portable instrument for time-domain near-infrared spectroscopy (TD-NIRS), we characterized healthy neonates’ brains in term of optical properties and hemodynamic parameters. In particular, we assessed the absolute values of the absorption and reduced scattering coefficients at two wavelengths, together with oxy-, deoxy- and total hemoglobin concentrations, and the blood oxygen saturation of the neonates’ brains. In this study, 33 healthy full-term neonates were tested, obtaining the following median values: 0.28 and $0.31\;{\mathrm{cm}}^{-1}$ for $\mu_{a}$ at 690 and 820 nm, respectively; 5.8 and $5.3\;{\mathrm{cm}}^{-1}$ for $\mu_{s}^{\prime }$ at 690 and 820 nm, respectively; 103  μM for $c_{O_{2}\mathrm{Hb}}$; 42.6  μM for $c_{\mathrm{HHb}}$; 146  μM for $c_{t\mathrm{Hb}}$; 72% for ${\mathrm{sO}}_{2}$. In general, the agreement of these values with the sparse existing literature appears not always consistent. These findings demonstrate the first measurements of optical properties of the healthy neonate brain using TD-NIRS and show the need for clarification of optical properties across methods and populations.

## 1 Introduction

Near-infrared spectroscopy (NIRS) is an optical technique offering a noninvasive and safe method to study the human brain, allowing both a functional approach and the evaluation of its optical (absorption and reduced scattering coefficients $\mu_{a}$ and $\mu_{s}^{\prime }$, respectively) or physiological baseline characteristics. Since Jöbsis^1^ demonstrated that, by exploiting the transparency of biological media to the near-infrared light, it was possible to assess the oxygenation of cerebral tissues, an increasing interest has been focused on the use of NIRS for adult and infant brain measurements.^2^ In fact, if one considers the hemoglobin as the main absorbing chromophore at the operating wavelengths, the information about oxy- and deoxyhemoglobin concentrations ($c_{O_{2}\mathrm{Hb}}$ and $c_{\mathrm{HHb}}$, respectively) inside a tissue can be easily derived from the measurement of its absorption properties. Furthermore, because the light diffuses inside tissues, measurements in reflectance configuration are possible, making feasible a flexible and experiment-designed configuration of optodes.

In most experiments presented in literature, the NIRS setup is based on a continuous wave (CW) approach. Despite some advantages of this method, the CW technique is intrinsically not able to retrieve separately the absorption and scattering properties of the investigated tissue: from CW data, it is possible to determinate only a tissue oxygenation index, which is the product $\mu_{a}\times \mu_{s}^{\prime }$, considered as a “scaled absolute absorption coefficient.”^3^ This limitation of CW approach can be overcome considering a multidistance approach without trivial preliminary calibration of the instrument.^4^ In general, a CW NIRS device cannot give information about the absolute values of the optical parameters, but it can detect only variations of the absorption properties of tissues. Then, the CW approach is appropriate to study the functional behavior of the brain, where experimental protocols are usually designed to detect hemodynamic changes. On the contrary, it is not suited for absolute quantification of the baseline optical characteristics without any assumption on $\mu_{s}^{\prime }$.

A second approach to NIRS is based on the use of light pulses hundreds of picoseconds broad.^5^ The time-domain (TD) approach to NIRS allows the reconstruction and the distribution of time-of-flight (DToF) of the detected photons. By exploiting proper physical models for photon migration,^6^ from the measured time-resolved reflectance (TRR) curves, it is possible to discriminate between tissue optical properties assessing the absolute values of $\mu_{a}$ and $\mu_{s}^{\prime }$.

A third approach is the frequency-domain (FD) NIRS, where the source is a radiofrequency modulated light, and the measures of the phase shift and light attenuation are considered. If a sufficient number of modulational frequencies are simultaneously used, in principle, the absolute optical properties of the investigated tissue can be retrieved, this approach being equivalent through a Fourier transformation to the TD-NIRS. However, this is not usually the case and the use of more than one modulational frequency has to be considered such as an exception.^7^

Application of NIRS is particularly suitable and attractive on infants first because of the safe, noninvasive, and wearable characteristics of this technique, as well as for its low cost and portability. In fact, NIRS can integrate or substitute other imaging modalities that are usually limited in studying neonates, children, or, in general, subjects without a full control of movements. Furthermore, the thinner skull thickness of infants’ heads with respect to adults improves the sensitivity on brain regions, making NIRS experiments on babies particularly successful. In particular, because the overall thickness of scalp and skull in neonates can be estimated of about 5 mm,^8^ assumptions on the head geometry can be simplified by modeling it as a homogeneous semi-infinite medium. This approximation is not valid for adults, where multilayered models have to be considered to account for the real structure of the head.^9^

Several functional experiments have been carried out on babies both for research or clinical activities, and they are mainly focused on their cognitive or behavioral development, using NIRS to measure tissue oxygenation associated with neural activity.^10^^–^^13^ Conversely, up to now, only few studies have been performed by NIRS about *in vivo* baseline optical or physiological properties of the neonate brain. This is mainly due to the fact that most of the commercially available systems for NIRS are based on CW technology, and thus present the limitations described above. For instance, the group of Zhao et al.^14^ used a FD-NIRS setup to measure baseline optical properties and, from them, they recovered the concentrations $c_{O_{2}\mathrm{Hb}}$ and $c_{\mathrm{HHb}}$ of the two hemoglobin species, oxy- and deoxyhemoglobin, respectively, the concentration $c_{t\mathrm{Hb}}$ of the total hemoglobin content, and the oxygen saturation ${\mathrm{sO}}_{2}$. A monitoring experiment on neonates about the brain development (in terms of $c_{t\mathrm{Hb}}$, ${\mathrm{sO}}_{2}$, blood volume, and cerebral metabolic rate of oxygen) was performed by Franceschini et al.^15^ and shows consistency with previous positron emission tomography and electroencephalogram studies. In a more recent paper by a Swiss group (see Ref. 16), they were studied, by exploiting a FD-NIRS system, the effects of the homogeneity assumption of infant head on the precision of hemoglobin concentration measurements. Baseline absorption and scattering parameters are also tabulated therein. A study of brain optical properties at different ages (from neonates to adults) and with different geometry models was conducted by a Harvard research group by using a FD system for NIRS.^9^

To our knowledge, only one study about the baseline characterization of neonate brain performed by TD-NIRS has been published in literature until now,^17^ where researchers provided data of $\mu_{a}$, $\mu_{s}^{\prime }$, ${\mathrm{sO}}_{2}$, and cerebral blood volume. They also studied a possible relationship between these variables and the neonate postconceptional age. However, the population of neonates they considered was undergoing neonatal intensive care. In this paper, we report about a study, where TD-NIRS is employed on healthy full-term neonates, in order to determine the brain optical properties $\mu_{a}$ and $\mu_{s}^{\prime }$, and hemodynamic parameters $c_{O_{2}\mathrm{Hb}}$, $c_{\mathrm{HHb}}$, $c_{t\mathrm{Hb}}$, and ${\mathrm{sO}}_{2}$. The ultimate goal of this study is to provide the scientific community with a further and numerically consistent dataset concerning the characterization of the neonate healthy brain from the optical, i.e., in the visible and near-infrared spectral range, point of view.

## 2 Materials and Methods

### 2.1 Subjects

Thirty-three healthy full-term neonates (13 male and 20 female, 3.2±0.9 days after birth) underwent the measurements at the Presidio Ospedaliero Universitario “Santa Maria della Misericordia” in Udine, Italy. All infants had an Apgar score higher than 7 at 1 and 5 min after birth and no cephalohematomas. Clinical details of neonates are summarized in Table 1. Written informed consent was signed by parents of every neonate prior to the enrollment, and the study was approved by the local ethics committee.

**Table 1:** Clinical details of the enrolled neonates. M: male; F: female; NA: not available; SD: standard deviation.

Neonate (#)	Gender	Age (days)	Gestational age (weeks)	Weight (kg)	Circumference head (cm)
1	M	4	39	3.328	35.0
2	F	4	39	3.086	34.3
3	M	5	41	3.474	35.2
4	M	2	39	3.458	36.0
5	F	4	38	3.416	34.4
6	M	3	39	3.430	33.5
7	M	4	39	3.273	34.5
8	M	3	40	3.700	35.5
9	M	3	40	3.208	34.2
10	M	5	38	3.182	35.0
11	F	3	41	3.034	32.5
12	F	2	40	4.078	35.0
13	F	5	37	3.336	35.0
14	F	4	39	2.736	33.0
15	F	3	39	2.790	32.6
16	F	4	39	3.188	34.5
17	F	3	39	2.716	33.0
18	F	3	39	2.890	32.5
19	F	4	38	3.184	32.5
20	F	2	39	3.314	34.0
21	F	4	41	3.268	35.0
22	F	3	40	3.262	34.2
23	F	4	38	2.462	34.0
24	M	2	38	3.402	35.0
25	F	2	38	3.500	33.0
26	M	2	39	3.233	34.5
27	M	2	40	3.374	35.0
28	F	2	38	3.360	34.0
29	M	3	NA	NA	NA
30	M	4	41	2.940	33.5
31	F	5	41	3.612	35.5
32	F	3	NA	NA	NA
33	F	3	40	3.000	35.0
Mean		3.30	39.23	3.233	34.22
SD		0.98	1.09	0.319	0.99

### 2.2 TD-NIRS Instrument

Measurements have been performed by the class I medical device for TD-NIRS developed at the Physics Department of Politecnico di Milano. The light sources are two pulsed diode lasers (Picoquant GmbH, Germany) operating at 690±10  nm and 820±10  nm, with a repetition rate of 80 MHz, pulse duration of about 100 ps, and average power less than 2 mW. The laser pulses are coupled into two multimode graded index fibers (50/125  μm core/cladding diameter) of different length and time multiplexed by means of a 2×2 fused fiber optics splitter (OZOptics, Canada), in order to create two independent sources. Then, a couple of 1×9 fiber optics switches (PiezoJena GmbH, Germany) are used to create up to 16 independent injection points (one channel in each optics switch is sacrificed to create a stopping channel). The light power delivered on the sample is far less than 1 mW, 15 times below the safety limit.

The detection of remitted photons, after propagation in the diffusive medium, is accomplished by four parallel and identical detection chains. Each chain presents the combination of a compact four-channel photomultiplier (Hamamatsu Photonics, Japan), a home-made amplifier with variable gain, and routing electronics (Becker&Hickl, Germany). All the photomultipliers are cooled by means of Peltier units and metal heat sink to reduce thermal noise. All the 16 detection channels are equipped with an independent variable attenuator in order to equalize the signal coming from different areas of the sample. The registration of the DToFs is performed by four time-correlated single-photon counting (TCSPC) boards (Becker&Hickl, Germany). More details about the instruments and their performances are described in Ref. 18.

**Fig. 1:**
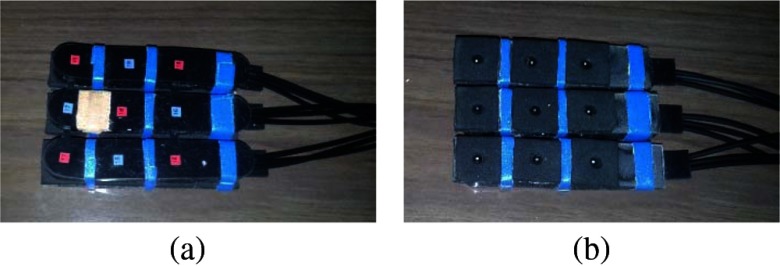
Silicon probe use for the measurements on the neonates’ heads. Red squares: injection points; blue squares: detection points. (a) External side and (b) surface in contact with the head (fiber tips are visible).

**Fig. 2:**
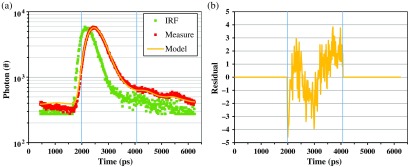
(a) It is reported a typical TRR curve (red squares) recorded at 690 nm with the TD-NIRS device, together with the corresponding IRF (green squares) and the model resulting from fitting procedure (yellow line). The FWHM of IRF is 640 ps, while the number of photons of the TRR curve is about $5\times 10^{5}$. The vertical light blue lines mark the range considered for the best fit of the model to the data: the retrieved optical properties in this case are $\mu_{a}=0.26\;{\mathrm{cm}}^{-1}$ and $\mu_{s}^{\prime }=7.7\;{\mathrm{cm}}^{-1}$. (b) The residuals of the fitting procedure are reported, calculated as $(O_{i}-M_{i})/\sqrt{M_{i}}$, where $O_{i}$ is the i’th observation and $M_{i}$ is the correspondent calculated model (see main text). From residuals, the reduced $\chi^{2}$ value of the fitting procedure can be calculated resulting in this case 3.22.

We note that the system can manage up to 16 injection points and up to 16 collection points. Furthermore, it can be used with different types of collecting fibers or fiber bundles. For the present experiment, we adapted the device in order to use as probes two soft black silicon cushions, into which small fiber bundles (1 mm in diameter), made of step-index optical fibers, for light delivery and detection, are embedded (see Fig. 1). These probes were light and comfortable, and the soft silicon cushions allowed an optimized contact with the infant scalp. Then a subset of five injection fibers and four detection channels were used in each silicon pad, providing 12 source–detectors pairs on each probe (overall 24 registered channels). The distance between detectors and receivers was 3 cm. In order to limit the temporal spread of light pulses due to their propagation into the fiber bundles, the latter ones were kept as short as possible, setting a final length of about 2.5 m. In this way, the instrument response function (IRF) of the device is on the order of 500 ps for FWHM (compare Fig. 2).

The source fibers were sequentially illuminated every 0.2 s in the left and right hemispheres in parallel, for an overall acquisition time of 1 s (1-Hz sampling rate for a full topographic map).

### 2.3 Measurement Protocol

Infants were tested in a quiet and dimmed room within their cribs. One silicon probe was placed over the right hemisphere, the other over the left one, in such a way to cover from the frontal area until the central areas of the neonates’ heads, centered on the perisylvian areas. The pads were fixed by a sterile bandage; moreover, an experimenter kept his hands on the probes for all the duration of the experiment in order to assure the best contact between scalp and optodes by following the infant’s head movements. The TD-NIRS device was positioned outside the room, and the acquisitions were observed via infrared video projection to an external monitor. A medical doctor was present in the room during the experiment to soothe the infant if necessary. After waiting a while for the complete adaptation of the infant to the environment, we proceeded with the acquisition of TD-NIRS signal, lasting 30 s.

The raw data collected in this study are not available to the community as open access data.

### 2.4 Data Analysis

Since in TCSPC technique, the Poisson noise, affecting the measures, is proportional to the square root of the acquired photon number, the TRR curves registered during the 30 s of acquisition were summed up for each measurement point, in order to increase the photon counts and thus improve the signal-to-noise ratio (SNR). Next, the obtained TRR curves were fitted using the solution of the diffusion equation for a semi-infinite homogenous diffusive medium obtained considering extrapolated boundary conditions,^6^ in order to retrieve the absorption and reduced scattering coefficients for each neonate’s brain at the two considered wavelengths. The refractive index of the brain tissue was fixed to 1.4. As a matter of fact, taking into account the very small thickness of scalp and skull in neonates (about 5 mm^8^) and the source–detector distance considered in the measurements (3 cm), the retrieved values for the absorption and reduced scattering coefficients are essentially those of the cerebral tissue.^19^ An exemplum of the results of the fitting procedure is reported in Fig. 2.

Once obtained the absorption coefficients at the two different wavelengths 690 and 820 nm, the absolute values of the oxy- and deoxyhemoglobin concentrations can be calculated by exploiting the Beer–Lambert’s law. If we assume that the oxy- and deoxyhemoglobin, together with water, are the main absorbers of the head tissues in the spectral range considered, then it results:

$$\left\{ \begin{array}{c} \mu_{a}^{690}=\varepsilon_{\mathrm{HHb}}^{690}\cdot c_{\mathrm{HHb}}+\varepsilon_{O_{2}\mathrm{Hb}}^{690}\cdot c_{O_{2}\mathrm{Hb}}+\varepsilon_{H_{2}O}^{690}\cdot c_{H_{2}O}\\ \mu_{a}^{820}=\varepsilon_{\mathrm{HHb}}^{820}\cdot c_{\mathrm{HHb}}+\varepsilon_{O_{2}\mathrm{Hb}}^{820}\cdot c_{O_{2}\mathrm{Hb}}+\varepsilon_{H_{2}O}^{820}\cdot c_{H_{2}O} \end{array},\right.$$ (1)

where $\mu_{a}^{\lambda }$ are the absorption coefficients at a given wavelength λ, $\varepsilon_{X}^{\lambda }$ are the extinction coefficients of the chromophores X (oxy- and deoxyhemoglobin and water) at a given wavelength λ, and $c_{X}$ are the concentrations of the chromophores X. We explicitly note that here we are considering the adult hemoglobin. Even if in neonates’ blood also the fetal hemoglobin is present, it rapidly declines in the first weeks of life in favors of the adult hemoglobin. Furthermore, the absorptivities of fetal and adult hemoglobin result indistinguishable in the spectral range of interest here, as demonstrated in Refs. 20 and 21. Then, in general, we will refer to the concentrations of the adult hemoglobin derivatives (that is oxy- and deoxyhemoglobin).

Next, we fixed the water concentration $c_{H_{2}O}$ to 90% and inverted the linear system [Eq. (1)] in order to get the oxy- and deoxyhemoglobin content, $c_{O_{2}\mathrm{Hb}}$ and $c_{\mathrm{HHb}}$, respectively.^22^ The values we used for the extinction coefficients of the chromophores are reported in Table 2.^23^

**Table 2:** Values for the extinction coefficients of the chromophores used for the inversion of Beer–Lambert’s law reported in Eq. (1).

Wavelength (nm)	$\varepsilon_{O_{2}\mathrm{Hb}}$ (${\mathrm{cm}}^{-1}\;\mu M^{-1}$)	$\varepsilon_{\mathrm{HHb}}$ (${\mathrm{cm}}^{-1}\;\mu M^{-1}$)	$\varepsilon_{H_{2}O}$ (${\mathrm{cm}}^{-1}$)
690	$6.36\cdot 10^{-4}$	$4.73\cdot 10^{-3}$	$4.72\cdot 10^{-3}$
820	$2.11\cdot 10^{-3}$	$1.60\cdot 10^{-3}$	$2.41\cdot 10^{-2}$

Finally, the concentration $c_{t\mathrm{Hb}}$ of the total hemoglobin and the oxygen saturation ${\mathrm{sO}}_{2}$ can be calculated as follows:

$$c_{t\mathrm{Hb}}=c_{\mathrm{HHb}}+c_{O_{2}\mathrm{Hb}},$$ (2)

$${\mathrm{sO}}_{2}=\frac{c_{O_{2}\mathrm{Hb}}}{c_{t\mathrm{Hb}}}.$$ (3)

## 3 Results and Discussion

Preliminarily, in order to have results as reliable as possible, we introduced two criteria for the inclusion of measurements in the following analysis.

First, we excluded the TRR curves for which the best fitting procedure with the theoretical model (see Sec. 2.4) ends with a figure of merit, i.e., the reduced $\chi^{2}$ value, larger than 10. In particular, in the least square curve fitting procedure adopted here, the reduced $\chi^{2}$ was calculated in the typical way:

$$\chi^{2}=\frac{1}{f}\sqrt{\overset{N}{\underset{i=1}{\sum }}\frac{{\left(O_{i}-M_{i}\right)}^{2}}{\sigma_{i}^{2}}},$$ (4)

where f are the degrees of freedom, i.e., the number N of observations minus the number of retrieved parameters, $O_{i}$ is the i’th observation, i.e., the number of photons measured in a given time channel (compare Fig. 2), $M_{i}$ is the correspondent calculated model, and $\sigma_{i}$ is the expected error of the observation $O_{i}$. Because in the TCSPC technique, measured data are affected by Poisson noise, we set $\sigma_{i}=\sqrt{M_{i}}$, where we consider the calculated value instead of the observed value because this guarantees a more stable fitting procedure at low counts.^24^ According to this definition, the expected value for the reduced $\chi^{2}$ is 1. However, we decide to tolerate reduced $\chi^{2}$ values up to 10: as an exemplum, for the fitting procedure reported in Fig. 2, the value of the reduced $\chi^{2}$ is 3.22.

Second, we assumed that TRR curves have an acceptable SNR only if they have at least $10^{4}$ counts. Indeed, the accuracy of the fitting procedure is within 5% also at this count level.^24^

**Fig. 3:**
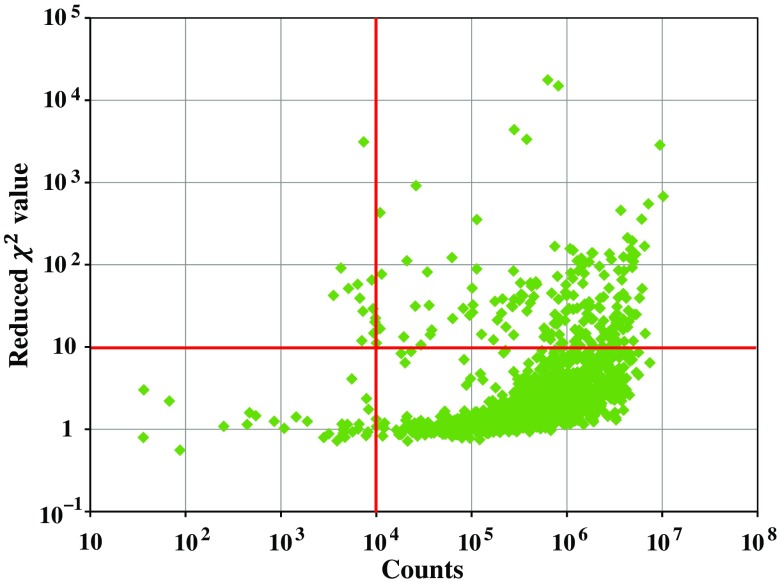
Scatter plot in logarithmic scale of reduced $\chi^{2}$ value obtained in the best fitting procedure as a function of the detected photon counts for each measured TRR curve. Red lines represent the thresholds set for data rejection.

In Fig. 3, it is reported for each measured TRR curve, the reduced $\chi^{2}$ value obtained in the best fitting procedure as a function of the counts.

We observe that the majority of the measurements (88% for both wavelengths) fulfill the inclusion criteria. In particular, for each of the subjects from 1 to 29, on average, 22.6±1.7 registered channels fulfill the inclusion criteria and, then, will be considered for the following analysis. On the contrary, the neonates 30, 31, 32, and 33 have been totally excluded from data analyses because only a few numbers of registered channels fulfill the inclusion criteria (only 7.5±4.2 for each of these subjects). This is mainly due to the presence of important movement artifacts during the measurements on these neonates.

**Fig. 4:**
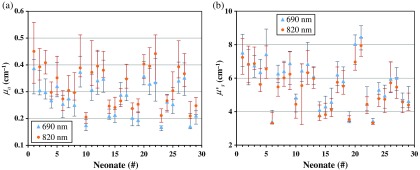
(a) Median values of absorption coefficient $\mu_{a}$ and (b) reduced scattering coefficient $\mu_{s}^{\prime }$, calculated for different neonates. Error bars represent the 25th and 75th percentile of the values distribution.

**Fig. 5:**
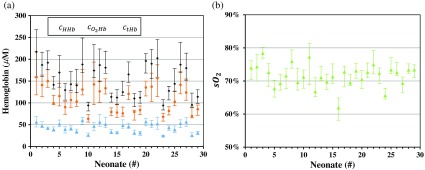
Median values of the (a) oxy-, deoxy- and total hemoglobin concentrations $c_{O_{2}\mathrm{Hb}}$, $c_{\mathrm{HHb}}$, and $c_{\mathrm{tHb}}$ and (b) the oxygen saturation ${\mathrm{sO}}_{2}$, calculated for different neonates. Error bars represent the 25th and 75th percentile of the values distribution.

As for the optical properties, in Fig. 4, they are reported as the median values of absorption and reduced scattering coefficients $\mu_{a}$ and $\mu_{s}^{\prime }$ of the neonates’ brains, calculated at two wavelengths by considering all the registered channels for each neonate. Furthermore, in Fig. 5, they are reported as the median values of the oxy-, deoxy- and total hemoglobin concentrations $c_{O_{2}\mathrm{Hb}}$, $c_{\mathrm{HHb}}$ and $c_{t\mathrm{Hb}}$, and the oxygen saturation ${\mathrm{sO}}_{2}$ of the neonates’ brains, calculated as in Fig. 4 for each neonate. In order to have an idea of the distribution of values registered on each neonate, in Figs. 4 and 5 the 25th and 75th percentile of such distribution are also reported as error bars. Even if the intrasubject variability, i.e., the variability among the 24 registered channels for each neonate, is important, then the intersubject variability appears prominent. In order to explain the observed intrasubject variability in the measured data, we can note that, besides the physiological variations among different positions of the neonates’ heads, an important role is probably played by the head curvature, which also varies and could affect the recovered optical properties in different ways. Furthermore, the fact that the probe has been held in position by the operator’s hands could have had some influence, too.

In order to make the dataset on neonates’ brains more usable, the values of optical properties $\mu_{a}$ and $\mu_{s}^{\prime }$ at two wavelengths, and of the hemodynamic parameters $c_{O_{2}\mathrm{Hb}}$, $c_{\mathrm{HHb}}$, $c_{t\mathrm{Hb}}$, and ${\mathrm{sO}}_{2}$, shown in Figs. 4 and 5, are reported in the Appendix (see Table 3). Here, we report the same parameters for the overall neonates’ population. In the format, “median [25th–75th percentile],” we obtained: 0.28 $[0.22-0.34]\;{\mathrm{cm}}^{-1}$ and 0.31 $[0.25-0.40]\;{\mathrm{cm}}^{-1}$ for $\mu_{a}$ at 690 and 820 nm, respectively; 5.8 [4.6–7.0] and 5.3 $[4.2-6.6]\;{\mathrm{cm}}^{-1}$ for $\mu_{s}^{\prime }$ at 690 and 820 nm, respectively; 103 [80.2−139]  μM for $c_{O_{2}\mathrm{Hb}}$; 42.6 [33.4−53.6]  μM for the $c_{\mathrm{HHb}}$; 146 [119−188]  μM for $c_{t\mathrm{Hb}}$; 72% [68%–75%] for ${\mathrm{sO}}_{2}$.

**Table 3:** Median values of optical and hemodynamic parameters for different subjects.

Neonate (#)	$\mu_{a}$ (${\mathrm{cm}}^{-1}$)		$\mu_{s}^{\prime }$ (${\mathrm{cm}}^{-1}$)		$c_{\mathrm{HHb}}$ (μM)	$c_{O_{2}\mathrm{Hb}}$ (μM)	$c_{t\mathrm{Hb}}$ (μM)	${\mathrm{sO}}_{2}$
	690 nm	820 nm	690 nm	820 nm				
1	0.39	0.45	7.52	7.24	56.36	158.88	216.91	0.74
2	0.31	0.39	6.86	6.83	47.68	140.94	186.95	0.74
3	0.30	0.41	6.75	6.87	42.40	150.04	192.16	0.78
4	0.27	0.30	6.35	5.63	39.37	99.51	141.37	0.73
5	0.32	0.35	7.42	6.55	52.12	115.68	169.53	0.68
6	0.25	0.27	3.40	3.32	39.88	90.44	129.49	0.69
7	0.27	0.30	6.27	5.48	41.90	106.70	142.95	0.72
8	0.25	0.30	6.41	6.02	34.58	103.16	140.10	0.76
9	0.37	0.39	6.89	6.24	59.86	130.70	188.14	0.70
10	0.17	0.21	4.83	4.44	26.32	63.70	94.48	0.71
11	0.31	0.37	6.45	5.56	46.73	142.60	174.32	0.77
12	0.34	0.40	6.84	6.32	56.21	126.51	186.72	0.67
13	0.35	0.38	6.10	5.98	51.39	133.72	180.84	0.71
14	0.21	0.25	4.10	3.74	33.50	79.36	113.84	0.70
15	0.21	0.24	4.30	3.82	32.15	77.95	112.35	0.71
16	0.29	0.27	4.57	4.12	48.83	76.00	124.81	0.62
17	0.29	0.35	6.21	5.76	46.27	120.68	165.32	0.73
18	0.20	0.24	5.81	5.52	31.62	79.34	110.19	0.69
19	0.19	0.25	3.53	3.54	31.76	83.74	111.94	0.73
20	0.36	0.40	8.04	6.97	57.14	135.35	196.25	0.70
21	0.33	0.40	8.46	7.95	51.77	137.24	189.82	0.73
22	0.34	0.44	4.46	4.44	52.53	156.69	202.58	0.75
23	0.17	0.21	3.43	3.32	24.84	68.18	94.21	0.72
24	0.26	0.27	5.30	4.77	43.70	81.79	127.54	0.66
25	0.25	0.30	4.95	4.74	38.86	102.66	143.73	0.73
26	0.34	0.39	5.96	5.72	53.34	141.48	187.69	0.73
27	0.35	0.37	6.02	5.46	56.72	123.92	179.74	0.69
28	0.17	0.21	4.68	4.58	25.77	69.86	95.34	0.73
29	0.21	0.25	4.63	4.40	31.42	85.55	114.05	0.73
Median (25th–75th)	**0.28**	**0.31**	**5.8**	**5.3**	**42.6**	**103**	**146**	**0.72**
	(0.22–0.34)	(0.25–0.40)	(4.6–7.0)	(4.2–6.6)	(33.4–53.6)	(80.2–139)	(119–188)	(0.68–0.75)

Now, we compare our dataset about optical properties and hemodynamic parameters of neonates’ brains with similar datasets present in scientific literature. First, we have to say that there is little about this issue. In particular, as we stated in Sec. 1, to our knowledge, a TD-NIRS instrumentation has been exploited in only one study about the baseline characterization of neonates’ brains.^17^ Even though therein the authors considered a population of neonates that underwent neonatal intensive care and the wavelength used was not exactly the same, it is anyway interesting to compare results we obtained with theirs. As for the reduced scattering coefficients, the values obtained here are compatible with results in Ref. 17. On the other hand, the values for absorption coefficients found in this work are larger by about 50% with respect to those found in Ref. 17. Consequently, we also observe here an overestimation for the hemoglobin content. Nevertheless, the blood oxygen saturation results are comparable in the two works.

In a few other papers in literature, the baseline characterization of neonates’ brains has been performed by exploiting a FD-NIRS.^14^^–^^16^^,^^25^ In particular, in Refs. 15, 16, and 25, only the hemodynamic parameters are provided. The total hemoglobin content reported in these works is about 50−60  μM, i.e., a value for $c_{t\mathrm{Hb}}$ that is lower with respect to what we reported here, and similar to what is reported in Ref. 17. On the other hand, again we observed comparable values for the blood oxygen saturation. In Ref. 14, they reported also the optical properties of the neonates’ brain tissue. In this case, the absorption and reduced scattering coefficients at two wavelengths, similar to those used here, are lower and larger, respectively, than the values reported in Table 3.

In summary, the values reported in Table 3 about the optical properties and the hemodynamic parameters of neonates’ brain are not always compatible to similar data reported elsewhere. In particular, we can state that the blood oxygen saturation is comparable with literature data, while the absorption coefficient of neonates’ brains reported here is in general overestimated with respect to literature data. We want to stress that literature data are in general few and the population of neonates considered in the different studies sometimes are not comparable: for example, in this paper, we considered only healthy neonates, while in the cited papers are reported results involving also neonates with some pathology. If we add that the majority of data on neonates’ brains have been obtained with a different technique, i.e., the FD-NIRS, we think that there is room for reporting also the dataset presented in this paper and obtained by exploiting a TD-NIRS instrumentation. We hope that further studies on this important issue will be performed also with different techniques, in such a way as to arrive at a reliable, consolidated, and widely accepted dataset about the optical properties and hemodynamic parameters of neonates’ brains.

## 4 Conclusions

In this paper, we determined optical properties and hemodynamic parameters of the brain of 33 full-term healthy neonates. An instrument for multichannel TD-NIRS was employed. By adopting fair exclusion criteria for the performed measurements, we obtained a reliable dataset that we compared with a similar dataset already reported in literature that used a different population (newborns in intensive care compared to healthy newborns in our dataset) and a different instrumental approach to NIRS (FD-NIRS as compared to TD-NIRS used here). We found consistencies in the measures of blood oxygenation but variations in the absorption coefficients. However, data concerning the optical characterization of the healthy neonates’ brains in the visible and near-infrared spectral range are few and, for this reason, not conclusive. The data presented here, from a large sample of healthy neonates, demonstrate the optical properties in a healthy newborn’s brain, but show that further research is necessary to clarify how these properties differ by the measurement techniques and population tested.

## Appendix: Neonates’ Dataset Details

In Table 3, we explicitly report the values of optical properties $\mu_{a}$ and $\mu_{s}^{\prime }$ at two wavelengths, and of the hemodynamic parameters $c_{O_{2}\mathrm{Hb}}$, $c_{\mathrm{HHb}}$, $c_{t\mathrm{Hb}}$, and ${\mathrm{sO}}_{2}$, already shown in Figs. 4 and 5. In this way, the neonates’ dataset will be more usable and comparisons with other similar datasets, already present in literature or that will be assessed in the future, more straightforward.
